# Did You Hear the One About the Doctor? An Examination of Doctor Jokes Posted on Facebook

**DOI:** 10.2196/jmir.2797

**Published:** 2014-02-13

**Authors:** Matthew A Davis, Carol Sue Haney, William B Weeks, Brenda E Sirovich, Denise L Anthony

**Affiliations:** ^1^The Dartmouth Institute for Health Policy and Clinical PracticeLebanon, NHUnited States; ^2^Institute for Quantitative Biomedical Sciences Graduate Program, Dartmouth CollegeHanover, NHUnited States; ^3^TolunaWilton, CTUnited States; ^4^Outcomes Group, VA Medical CenterWhite River Junction, VTUnited States; ^5^Geisel School of Medicine at DartmouthHanover, NHUnited States; ^6^Department of Sociology, Dartmouth CollegeHanover, NHUnited States

**Keywords:** physicians, physician-patient relations, social networking, humor

## Abstract

**Background:**

Social networking sites such as Facebook have become immensely popular in recent years and present a unique opportunity for researchers to eavesdrop on the collective conversation of current societal issues.

**Objective:**

We sought to explore doctor-related humor by examining doctor jokes posted on Facebook.

**Methods:**

We performed a cross-sectional study of 33,326 monitored Facebook users, 263 (0.79%) of whom posted a joke that referenced doctors on their Facebook wall during a 6-month observation period (December 15, 2010 to June 16, 2011). We compared characteristics of so-called jokers to nonjokers and identified the characteristics of jokes that predicted joke success measured by having elicited at least one electronic laugh (eg, an LOL or “laughing out loud”) as well as the total number of Facebook “likes” the joke received.

**Results:**

Jokers told 156 unique doctor jokes and were the same age as nonjokers but had larger social networks (median Facebook friends 227 vs 132, *P*<.001) and were more likely to be divorced, separated, or widowed (*P*<.01). In 39.7% (62/156) of unique jokes, the joke was at the expense of doctors. Jokes at the expense of doctors compared to jokes not at the expense of doctors tended to be more successful in eliciting an electronic laugh (46.5% vs 37.3%), although the association was statistically insignificant. In our adjusted models, jokes that were based on current events received considerably more Facebook likes (rate ratio [RR] 2.36, 95% CI 0.97-5.74).

**Conclusions:**

This study provides insight into the use of social networking sites for research pertaining to health and medicine, including the world of doctor-related humor.

## Introduction

Laughter is the best medicine, as the saying goes. Sociologists identify humor as a social phenomenon embedded in interaction [1] that can affirm conventional views about the world [2], highlight status differences within or outside of a group [3,4], enhance social bonds [5,6], or relieve stress [7,8]. It may be the latter effect that underlies the saying about laughter as medicine, but joking about medical care or practitioners may have other implications. Scholars have worried about the declining status and authority of physicians for the past 3 decades or more [9-11]. Although doctor jokes have been around since ancient times (eg, McDonald [12]), joking about doctors may contribute to this decline. Other evidence suggests declining career satisfaction among physicians [13,14] as a possible result of this declining status.

Although it may be difficult to know if doctors think doctor jokes are funny, we do know that generally people tell jokes with the intention of amusing others [1] and this may be accompanied by a complex range of motivations, from the expression of disagreement or dissatisfaction to an indication of endearment and friendship [15]. Freud argued that humor was a socially acceptable form of aggression in modern life, particularly when directed toward high status or powerful others [16]. Research suggests that people use and appreciate humor when the target of the joke is from a group different than their own [3,4], which may both enhance cohesion within a group [5] and relieve tension or stress [17] producing the stated “medicinal” effect of laughter. Given the relatively high status of physicians in society, it makes sense they would be the target of jokes. In addition, because Americans are generally dissatisfied with their health care system [18], joking about doctors and medicine may provide a socially acceptable way to express that dissatisfaction. Joking about medicine is also likely to be an important coping mechanism for patients facing serious illness [19].

Other evidence suggests that higher status group members or those who aspire to higher status are more likely to engage in joking behavior, particularly in status-differentiated groups [20]. In addition, there is a relationship between whether the joker himself laughs first, the number of “audience” members, and how much others laugh at the joke [21]. According to Glenn [21], in groups of 2 or larger it is not typical for the joker to laugh first (unlike in dyads).

Although we seem to know more than one might think about joking and jokers, there is relatively little research on humor in spontaneous conversation [20-22]. This is likely because of the difficulty in capturing spontaneous conversations for analysis. Today, however, a great deal of social interaction occurs online in social networking sites, such as Facebook. Currently Facebook has 874 million active users worldwide, communicating in 70 different languages [23]. Although written Facebook conversations are not the same as in-person interactions, we sought to eavesdrop on casual interactions occurring on Facebook to examine jokes about doctors. Therefore, we performed the first study of social networking site conversations pertaining to health and medicine to examine the prevalence, characteristics, and success of doctor jokes posted on Facebook.

## Methods

### Ethics Approval

The Committee for the Protection of Human Subjects at Dartmouth College reviewed the study protocol and granted this study an expedited Institutional Board Review.

### Sample

To obtain information on doctor jokes posted on Facebook, we used data from the Harris Interactive Research Lifestreaming Panel. Upon agreeing to become a Lifestreaming Panel participant, these individuals gave Harris permission to record their private conversations on social networking sites such as Facebook and Twitter. Although both Facebook and Twitter could potentially be used to study jokes about doctors, we were granted access specifically to the Lifestreaming Panel’s Facebook data. Lifestreaming Panel participants are paid the sum of US $1.00 initially, and they are then eligible to participate in future surveys and activities in which they can receive other payments. Harris collects data from Lifestreaming Panel participants’ social networking sites as well as information on the participants’ characteristics. Harris can search Lifestreaming Panel participants’ Facebook walls using algorithms based on keywords. As of June 2011 (the time of our data collection), there were 33,326 adult Lifestreaming Panel participants.

**Figure 1:**
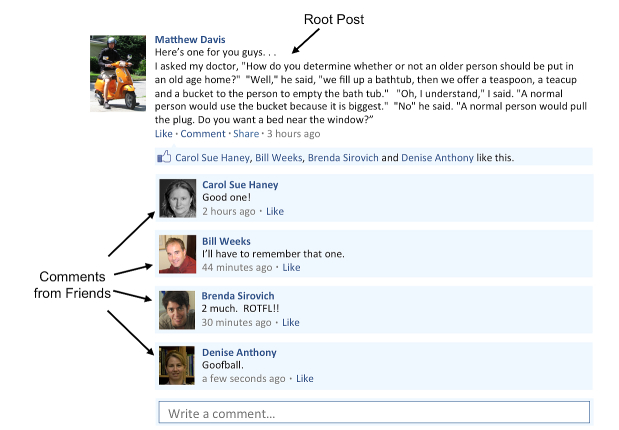
Fictional example of conversation thread on a Facebook wall.

On Facebook, each user creates a profile and has a personal “wall,” a place for conversing with others. For the purposes of this study, we defined a *conversation thread* as starting with a root post on a user’s wall by either the user or by the user’s “friend,” and including others’ follow-up comments. A fictional example of a conversation thread in which a doctor joke is mentioned can be found in Figure 1 (note: the authors are used to represent Facebook users to protect the identify of study participants).

### Identification of Doctor Jokes

**Figure 2:**
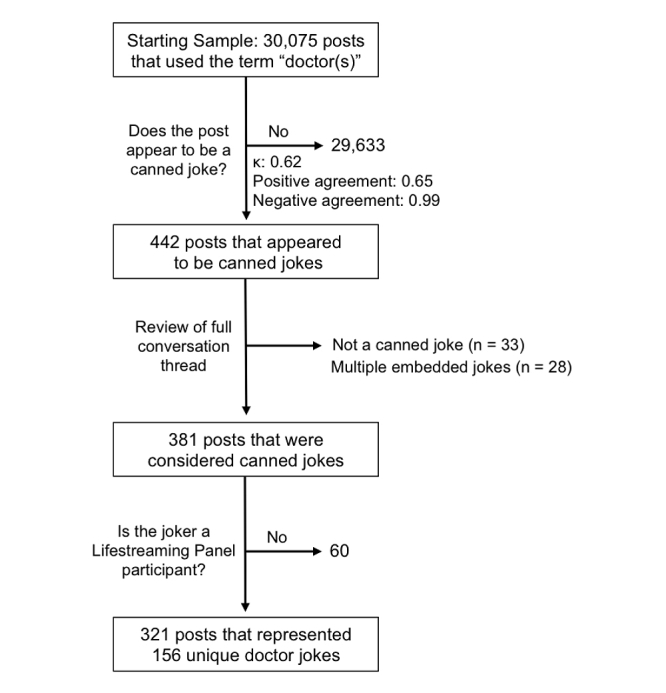
Flow diagram for study inclusion of doctor jokes.

Using Harris Interactive Research Lifestreaming Panel Facebook data from December 15, 2010 to June 16, 2011, we identified 30,075 individual posts that included the term “doctor(s)” (Figure 2). Because posts were identified first by the keyword doctor(s) and then attached to full Facebook wall threads, 60 doctor jokes were made by Facebook users not part of the Lifestreaming Panel (these individuals posted on a Lifestreaming Panel participant’s Facebook wall) and were therefore excluded from our study.

To identify jokes, 2 independent coders read each post that included the term doctor(s) in search of jokes. For practical reasons, coders were asked to only identify what they considered to be canned jokes. A typical canned joke contains an introduction followed by a punch line, and can stand alone as a humorous statement, free from context [24]. Coders identified 442 posts that contained potential canned jokes. Differences in coding were reconciled by a third reviewer. Because Cohen’s kappa statistic can underestimate the interreviewer agreement when analyzing rare events, we also calculated the positive and negative agreement between reviewers [25,26]. The kappa statistic, positive, and negative agreement for initial agreement between reviewers was 0.62, 0.65, and 0.99, respectively (Figure 2).

Three study investigators then read all 442 potential jokes and excluded those that were not canned jokes or that contained multiple embedded jokes. Differences were discussed and resolved by consensus. The process resulted in the identification of 321 posted doctor jokes that represented 156 unique canned jokes (some jokes were repeated). Selected examples of the jokes identified can be found in Table 1. We included doctor jokes in our sample whether or not the joke received follow-up posts from the social network.

**Table 1:** Selected examples of Facebook jokes made at the expense of doctors.^a^

Example number		Joke^b^
**Medical doctors or the medical profession**		
	(1)	Doctor, you told me I have a month to live and then you sent me a bill for $1000! I can’t pay that before the end of the month! Okay, says the doctor, you have 6 months to live ;)
	(2)	Here’s a question for you: What do you call a doctor who finishes last in his medical school class? Answer: Doctor.
	(3)	An old preacher was dying. He sent a message for his doctor and his lawyer to come. When they arrived, the preacher held out his hands and motioned for them to sit, one on each side of his bed. The preacher grasped their hands, sighed contentedly, smiled, and stared at the ceiling. For a time, no one said anything. Both the doctor and lawyer were touched and flattered that the preacher would ask them to be with him during his final moments. They were also puzzled; the preacher had never given them any indication that he particularly liked either of them. They both remembered his many long, uncomfortable sermons about greed, covetousness, and avaricious behavior that made them squirm in their seats. Finally, the doctor said, “Preacher, why did you ask us to come?” The old preacher mustered up his strength, then said weakly, “Jesus died between 2 thieves and that’s how I want to go.”
	(4)	Frank is recovering from day surgery when a nurse asks him how he is feeling. “I’m fine but I didn’t like the 4-letter-word the doctor used in surgery,” he answered. “What did he say?” asked the nurse. “Oops!”
	(5)	Three out of 4 doctors recommend another doctor.
**Health care system**		
	(1)	Two patients limp into 2 different medical clinics with the same complaint. Both have trouble walking and appear to require a hip replacement. The first patient is examined within the hour, is x-rayed the same day, and has a time booked for surgery the following week. The second calls his family doctor after waiting 3 weeks for an appointment, then waits 8 weeks to see a specialist, then gets an x-ray, which isn’t reviewed for another week, and finally has his surgery scheduled for a month from then. Why the different treatment for the 2 patients? The first is a Golden Retriever. The second is a senior citizen :)
	(2)	A doctor says to a patient, “We’ve run every test we can think of and the results show you’re out of money.”
**Poking fun at doctor’s advice or the doctor-patient relationship**		
	(1)	My doctor told me to walk 5 kilometers a day. It’s been 5 days and I am 25 kilometers from home and don’t know how to get back! Haha.
	(2)	So the doctor says, “Take your clothes off and stick your tongue out the window.” I asked him, “What will that do?” The doctor says, “I’m mad at my neighbor.”
	(3)	A woman told her doctor, “I’ve got a bad back.” The doctor said, “It’s just old age.” The woman said, “I want a second opinion.” “OK,” the doctor said. “You’re ugly too.”

^a^Jokes at the expense of doctors (ie, doctors are the butt of the joke) include jokes about medical physicians or the medical profession, the health care system, and poking fun at advice from doctors or the patient-doctor relationship.

^b^We corrected minor typographical errors and misspellings to improve readability and further de-identify study participants. Emoticon translations: :) smiley face; ;) smiley face with clever wink.

### Measures

#### Characteristics of Jokers

We operationally defined a Lifestreaming Panel participant who posted a doctor joke during the 6-month time period as a joker. We compared sociodemographic characteristics for jokers to nonjokers (participants who did not post a doctor joke on their Facebook wall during the 6-month observation period) and used the total number of Facebook friends at the time of data collection as the size of the participant’s social network. Because of the skewed distribution, we report the median number of friends, as well as number of Facebook fan pages and groups to which the participants belonged.

#### Characteristics of Doctor Jokes

We categorized each joke into 1 of 2 groups: those that were made at the expense of doctors (defined as jokes in which medical doctors or the medical profession, doctors’ advice, the doctor-patient relationship, or the health care system were the butt of the joke) and those in which this was not the case. We also determined whether or not the joke was a pun (ie, dependent on multiple definitions of a word), used dirty humor (defined as including foul language, sexual content, racism, degradation of women, reference to human waste, or that were otherwise in poor taste), referred to popular media or culture (television, movies, comedians, or advertising), or related to either politics or current events.

We estimated joke effectiveness in 2 different ways. First, we determined whether the joke received at least one electronic laugh (from a person other than the joker) anywhere in the response from the social network. We operationally defined an electronic laugh as containing one of the following responses: laughing out loud (LOL), rolling on the floor laughing (ROTFL), or an interjection (eg, “baha” or “haha”). For the 11 jokes in which an electronic laugh was included with root post by the joker, it was not considered evidence for (or against) a joke’s success [21].

During the course of the 6-month data collection period, the Facebook feature known as the “like” button that allows members of a network to show enthusiasm for a particular post became popular. As a secondary measure of joke success, we collected the total number of Facebook likes for the 225 jokes in our data that appeared after Harris began collecting data on this feature.

### Analyses

We used Stata version 13.0 statistical software (StataCorp LP, College Station, TX, USA) for all analyses. The chi-square test for categorical variables and the *t* test for continuous variables were used to compare sociodemographic characteristics of jokers to nonjokers, except for measures of Facebook activity (ie, social network size and the number of fan and group pages to which a participant belonged), which we compared by using a Mann-Whitney test. Missing values were assigned a category for the comparison of characteristics between jokers and nonjokers for categorical variables.

Because some jokes were repeated in our sample, we used generalized linear mixed-effects models that were fit to allow each joke to have a random intercept. Fixed effects in our models included the characteristics of the joke and the covariates for age, sex, and network size as a categorical variable (ie, small vs medium or large social network). To determine if specific characteristics of jokes predicted the success of a joke, we assumed a binomial distribution for our models in which the dependent variable was whether or not the joke received at least one electronic laugh (yes vs no), and a Poisson distribution for our models in which the total number of likes was the dependent variable.

## Results

### Characteristics of Jokers

Among the 33,326 Facebook users in the Lifestreaming Panel, 29.99% (9994/33,326) mentioned the word doctor during the 6-month observation period, but only 263 (0.79%) posted a doctor joke. Jokers varied by US region (*P*<.001) and were more heavily represented in the Northeast (the region with the highest density of physicians per capita) [27] and the South (Table 2).

Jokers differed from the typical Lifestreaming Panel participant in several ways: they were less educated (eg, 16.0% of jokers reported having graduate training or an advanced degree whereas 20.38% of nonjokers did, *P*=.02) and they were more likely to be divorced, separated, or widowed (vs married, *P*<.01). Jokers had larger social networks than nonjokers: jokers had a median of 227 (IQR 138-369) Facebook friends compared to 132 (IQR 56-270) among nonjokers (*P*<.001).

**Table 2:** The characteristics of study participants according to joker status.

Sociodemographic characteristics		Joker status^a^		*P* ^b^
		Joker (n=263)	Nonjoker (n=33,063)	
**US region, n (%)**				<.001
	Northeast	55 (20.9)	5911 (17.88)	
	Midwest	59 (22.4)	8,575 (25.94)	
	South	98 (37.4)	11,249 (34.02)	
	West	38 (14.5)	6,826 (20.65)	
	Unknown	13 (4.9)	502 (1.52)	
Age, mean (SD)		44.9 (0.7)	44.76 (0.08)	.89
**Sex, n (%)**				.84
	Male	90 (34.2)	11,113 (33.61)	
	Female	173 (65.8)	21,950 (66.39)	
**Race/ethnicity, n (%)**				.16
	Hispanic	6 (2.3)	1281 (3.87)	
	Non-Hispanic White	216 (82.1)	26,764 (80.95)	
	Non-Hispanic Black	11 (4.2)	1657 (5.01)	
	Other or multiple races	19 (7.2)	2615 (7.91)	
	Unknown	11 (4.2)	746 (2.26)	
**Marital status, n (%)**				<.01
	Married	118 (44.9)	15,268 (46.18)	
	Never married	62 (23.6)	8689 (26.28)	
	Divorced, separated, or widowed	56 (21.3)	4554 (13.77)	
	Unknown	27 (10.3)	4552 (13.77)	
**Education, n (%)**				.02
	High school graduate or less	45 (17.1)	4937 (14.93)	
	Some college or associate’s degree	117 (44.5)	13,145 (39.76)	
	College degree	56 (21.3)	6732 (20.36)	
	Graduate training or advanced degree	42 (16.0)	6739 (20.38)	
	Unknown	3 (1.1)	1510 (4.57)	
**Self-reported annual earnings (US$), n (%)**				.08
	<35,000	96 (36.5)	9918 (30.00)	
	35,000-74,999	78 (29.7)	11,094 (33.55)	
	≥75,000	62 (23.6)	9096 (27.51)	
	Unknown	27 (10.3)	2955 (8.94)	
**Facebook activity, median (IQR)**				
	Friends	227 (138-369)	132 (56-270)	<.001
	Fan pages	128 (57-260)	48 (16-117)	<.001
	Groups	20 (9-36)	7 (2-19)	<.001

^a^Based on data from a 6-month observation period (from December 15, 2010 to June 16, 2011). Joker: posted a doctor joke; nonjoker did not post a doctor joke.

^b^Chi-square test used in comparison of proportions, *t* test used in comparison of means, and Man-Whitney test used for numbers of friends, fan pages, and groups on Facebook.

### Doctor Jokes

Of the 156 unique jokes, 112 (71.8%) appeared only once in our data. Of the jokes that were repeated, 2 jokes were particularly popular and were repeated approximately 30 times.

Among the 156 unique doctor jokes, the joke was made at the expense of doctors (and/or the health care system) in approximately half (62/156, 39.7%). In addition, 25.0% (39/156) of jokes relied on dirty humor, 19.9% (31/156) were puns, 14.1% (22/156) pertained to popular culture and media, and 5.8% (9/156) related to current events and politics.

### Joke Success

Approximately half of all jokes posted (133/321, 41.4%) received electronic laughter. Jokes made at the expense of doctors were more likely to receive electronic laughter although this did not reach statistical significance (46.5% vs 37.3%, *P*=.09). The marginal trend (OR 1.46, 95% CI 0.94-2.29) toward a higher rate of electronic laugher in response to jokes at doctors’ expense was only slightly attenuated after adjusting for age, sex, and social network size (Table 3). The likelihood of generating an electronic laugh was not dependent on other joke characteristics in both our univariate and adjusted models.

The median number of Facebook likes for doctor jokes was 2 (IQR 0-19). Ironically, the joke with the greatest number of Facebook likes (49 total likes from a network of 253 friends) was a “doctor, priest, lawyer” joke in which lawyers were the butt of the joke.

We observed similar associations between the characteristics of jokes and receiving Facebook likes as a measure of joke success (Table 3 vs Table 4). Jokes that were at the expense of doctors received nearly 50% more likes (rate ratio [RR] 1.48, 95% CI 0.96-2.27) in our adjusted model (Table 4). However, jokes that used what we deemed dirty humor were actually less likely to receive Facebook likes, whereas jokes that were based on current events/politics received more than double the amount of Facebook likes (RR 2.36, 95% CI 0.97-5.74; *P*=.06).

**Table 3:** Univariate and adjusted odds ratios (OR) from mixed models (n=321 jokes) for the association between joke characteristics and elicitation of electronic laugher from social network.

Joke characteristic	Univariate		Adjusted^a^	
	OR (95% CI)	*P*	OR (95% CI)	*P*
At the expense of doctors^b^	1.46 (0.94, 2.29)	.10	1.43 (0.91, 2.26)	.12
Dirty humor	1.22 (0.63, 2.35)	.56	1.11 (0.57, 2.16)	.76
Pun	0.99 (0.57, 1.74)	.98	1.01 (0.58, 1.77)	.97
Based on popular culture	0.82 (0.36, 1.86)	.64	0.79 (0.35, 1.81)	.58
Based on current events/politics	1.85 (0.52, 6.64)	.34	1.73 (0.48, 6.20)	.40

^a^Adjusted for age (continuous, years), sex, and network size (categorical, 0-171 vs 172-293 or ≥294 friends).

^b^Jokes at the expense of doctors (ie, doctors are the butt of the joke) include jokes about medical physicians or the medical profession, the health care system, and poking fun at advice from doctors or the patient-doctor relationship.

**Table 4:** Univariate and adjusted rate ratios (RR) from mixed models (n=225 jokes) for the association between joke characteristics and total Facebook likes from social network.

Joke characteristic	Univariate		Adjusted^a^	
	RR (95% CI)	*P*	RR (95% CI)	*P*
At the expense of doctors^b^	1.48 (0.96, 2.27)	.08	1.48 (0.96, 2.27)	.08
Dirty humor	0.62 (0.36, 1.08)	.09	0.62 (0.36, 1.09)	.10
Pun	1.13 (0.65, 1.97)	.65	1.15 (0.67, 2.00)	.62
Based on popular culture	0.62 (0.32, 1.18)	.14	0.62 (0.32, 1.20)	.16
Based on current events/politics	2.32 (0.96, 5.62)	.06	2.36 (0.97, 5.74)	.06

^a^Adjusted for age (continuous, years), sex, and network size (categorical, 0-171 vs 172-293 or ≥294 friends).

^b^Jokes at the expense of doctors (ie, doctors are the butt of the joke) include jokes about medical physicians or the medical profession, the health care system, and poking fun at advice from doctors or the patient-doctor relationship.

## Discussion

### Principal Results

To our knowledge, this is the first study to use actual Facebook conversations to examine doctor-related humor. Overall, we found a low prevalence of doctor jokes on Facebook and relatively few Facebook users posting jokes about doctors (and the health care system in general). Interestingly, those who posted a doctor joke were more likely to be divorced, separated, or widowed, and to have larger social networks (ie, more friends on Facebook). Given the previous findings that people who want to improve their social status are more likely to joke, it may be that divorced, separated, or widowed Facebook users tell doctor jokes to appeal to a potential partner but, of course, we cannot distinguish the reason from these data. Although initially it appeared that poking fun at doctors (as compared to doctor jokes that were not made at the expense of physicians) led to more successful jokes (in generating electronic laughs and the total number of Facebook likes), such findings were not statistically significant. In regards to Facebook likes, jokes based on current events and politics appeared to receive greater response from an individual’s social network, whereas dirty humor jokes received fewer likes.

We also observed that although most jokes appeared only once in our data, a few jokes were repeated many times. Based on our qualitative review of these more highly repeated jokes, they differed little from other jokes in our study. Although we are not able to determine the reason, these findings demonstrate how certain ideas can spread rapidly throughout social networks [28].

### Comparison With Prior Work

In recent years, medicine has not been immune to the impact of social networking sites, and there is growing interest in social networking sites among physicians and biomedical researchers. Social networking sites may have important applications for studying social interaction and communication related to health and medicine [29,30]. The medical community has largely focused on discussing the ethics of doctor-patient interaction on social networking sites [31-37] and professionalism of younger practitioners’ exposure via social networking sites [38-44]. Only more recently has interest emerged in using social networking sites to employ health interventions [45,46] and to identify certain health behaviors [47-49]. To date, there have been few empirical studies in the biomedical literature that examined conversations on social networking sites in nonpatient population groups.

Although our study examined doctor jokes posted on Facebook and does not represent a comprehensive analysis of public opinion of the medical profession and health care, our analyses are among the first to examine actual social networking site conversations [50]. Primary analysis of Facebook conversations could provide researchers the ability to examine certain health behaviors and popular opinion pertaining to health and medicine. Furthermore, analysis of social media conversations on a larger scale could have important uses, such as studying US public opinion regarding national health policy, developing new methods for public health surveillance, and for sociological study to understand social support for illness in virtual settings. However, as our study demonstrates, conversations from social media sites contain a mixture of both relevant and (depending on the use) potentially irrelevant material.

### Limitations

Our study has several limitations that must be acknowledged. First, considering that Lifestreaming Panel participants gave permission to have their Facebook data recorded, the potential for selection bias cannot be ruled out. Second, we used the term “doctor(s)” to identify posts pertaining to medical physicians, which may underestimate the total conversations pertaining to physicians, medical practitioners, or the medical profession. Given the casual nature of Facebook, we thought that the term doctor would be used more commonly than a more formal term such as “physician.” Finally, we identified potential jokes for analysis based on our definition of what constituted a joke in Facebook posts; others might have defined jokes differently than we did.

### Conclusions

Despite the inherent limitations of our research, this study demonstrates the potential of using social networking sites for research on health and medicine. The adoption of social networking has resulted in growing interest in using outlets such as Facebook and Twitter in creative ways. In this study, we demonstrate how actual data from Facebook conversations can be used to study doctor-related humor. In addition to serving as an example, this study highlights some of the practical considerations regarding the analysis of data from social networking sites.

## Abbreviations

LOL: laughing out loud
ROTFL: rolling on the floor laughing
RR: rate ratio
